# Supplementary material for: Urinary prostaglandin E2 as a biomarker for recurrent UTI in postmenopausal women
Source: Life Sci Alliance. 2021 May 6;4(7):e202000948. doi: 10.26508/lsa.202000948 (PMC8200289; doi:10.26508/lsa.202000948)
Supplement: Supplementary file 1 [file LSA-2020-00948_TableS1.docx]

**Table S1. Statistical analysis for the comparison of clinical variables between No Never, Remission and Relapse groups.** BMI (body mass index), AODM: adult-onset diabetes mellitus. EHT: Estrogen hormone therapy. NSAID: Nonsteroidal anti-inflammatory drugs. NSAID (0 = No NSAID, 1 = NSAID use), NSAID (0 = No NSAID, 1= Non-selective NSAID, 2 = Selective NSAID use). PVR: Post void residual. Sx. incontinence: prior surgery for incontinence or prolapse.

| Group Variable (Cohort 3) | Test | *p* value |
| --- | --- | --- |
| Race | Ordinal Logistic  Regression (OLR) | 0.986 (Caucasian),  0.349 (Hispanic),  0.591 (African American) |
| Gravidity | Chi-Square | 0.3516 |
| Parity | Chi-Square | 0.3007 |
| Diabetes (AODM) | OLR | 0.091 |
| Prolapse | OLR | 0.712 |
| Sx. Incontinence | Chi-Square | 0.3903 |
| Smoker | OLR | 0.344 |
| NSAID (0,1) | Chi-Square | 0.0723 |
| NSAID (0,1,2) | OLR | 0.398 (Non-Selective),  0.414 (Selective) |
| EHT | OLR | 0.043 |
| PGE_2_ (pg/ml) | Kruskal-Wallis | < 0.001 |
| Cr (µg/ml) | Kruskal-Wallis | 0.2076 |
| PGE_2_/Cr (pg/µg) | Kruskal-Wallis | <0.001 |
| Age (yrs) | ANOVA | 0.0953 |
| BMI (kg/m^2^) | Kruskal-Wallis | 0.035 |
| PVR | Kruskal-Wallis | 0.0874 |
| pH | Kruskal-Wallis | 0.2312 |
