# Supplementary material for: Urinary prostaglandin E2 as a biomarker for recurrent UTI in postmenopausal women
Source: Life Sci Alliance. 2021 May 6;4(7):e202000948. doi: 10.26508/lsa.202000948 (PMC8200289; doi:10.26508/lsa.202000948)
Supplement: Supplementary file 2 [file LSA-2020-00948_TableS2.docx]

**Table S2. Statistical analysis for the comparison of clinical variables between rUTI and control groups.** Comparison of clinical variables between active rUTI (Relapse) and control (Never+Remission) groups. BMI (body mass index), AODM: adult-onset diabetes mellitus. EHT: Estrogen hormone therapy. NSAID: Nonsteroidal anti-inflammatory drugs. NSAID (0 = No NSAID, 1 = NSAID use), NSAID (0 = No NSAID, 1= Non-selective NSAID, 2 = Selective NSAID use). PVR: Post void residual. Sx incontinence: prior surgery for incontinence or prolapse.

| Group Variable  (rUTI) | Test | *p* value |
| --- | --- | --- |
| Race | Fisher Exact Test | 0.1742 |
| Gravidity | Chi-Square | 0.5919 |
| Parity | Chi-Square | 0.4757 |
| Diabetes (AODM) | Chi-Square | 0.5188 |
| Prolapse | Fisher Exact Test | 0.7446 |
| Sx. Incontinence | Chi-Square | 0.5076 |
| Smoker | Fisher Exact Test | 0.5483 |
| NSAID (0,1) | Chi-Square | 0.0895 |
| NSAID (0,1,2) | Chi-Square | 0.0895 |
| EHT | Chi-Square | 0.0125 |
| PGE_2_ (pg/ml) | Kruskal-Wallis | < 0.001 |
| Cre (µg/ml) | Kruskal-Wallis | 0.1118 |
| PGE_2_/Cr (pg/µg) | Kruskal-Wallis | < 0.001 |
| Age (yrs) | T-test | 0.0356 |
| BMI (kg/m^2^) | Kruskal-Wallis | 0.047 |
| PVR | Kruskal-Wallis | 0.0561 |
| pH | Kruskal-Wallis | 0.0905 |
